# Anxiolytic Effect of Two Tobacco Essential Oils (*Nicotiana tabacum* Linn.) on Mice

**DOI:** 10.3390/molecules26144171

**Published:** 2021-07-09

**Authors:** Danqing Xie, Lei Yao, Yan Huang, Shuaifan Wu, Li Ma, Yuhong Li, Wencui Wang

**Affiliations:** 1 Department of Landscape Architecture, School of Design, Shanghai Jiao Tong University, 800 Dong Chuan Road, Shanghai 200240, China; xdairyqueen@sjtu.edu.cn (D.X.); yaolei@sjtu.edu.cn (L.Y.); malimali2006@sjtu.edu.cn (L.M.); 2 R&D Center for Aromatic Plants, Shanghai Jiao Tong University, 800 Dong Chuan Road, Shanghai 200240, China; 3 Huabao Flavours & Fragrances Co., Ltd., 1299 Yecheng Road, Shanghai 201821, China; huangyan@hbflavor.com (Y.H.); wushuaifan@163.com (S.W.)

**Keywords:** tobacco leaf, essential oil, anti-anxiety effect, behavioral tests, salivary corticosterone

## Abstract

Tobacco (*Nicotiana tabacum* Linn.) is a famous traditional herb used in folk medicine. The essential oils of tobacco have been demonstrated in modern studies to possess antioxidant, anti-inflammatory, and neuroprotective properties, while its anxiolytic effect has not been reported. The purpose of this study was to evaluate the anxiolytic effect of Yunnan tobacco essential oil (YTO) and Zimbabwe tobacco essential oil (ZTO) on mice. The constituents of YTO and ZTO were analyzed by GC/MS. The anxiolytic effect of YTO and ZTO (0.1%, 1%, and 10%, *v*/*v*) on male ICR mice was evaluated in the light–dark box test (LDB) and the elevated plus maze test (EPM) test via inhalation and transdermal administration. After the behavioral tests, salivary corticosterone levels in mice were measured. The behavioral analysis showed that the administration of both YTO and ZTO elevated the time that the mice spent in the light chamber in the LDB test compared to the untreated control. In the EPM test, YTO and ZTO increased the time spent in open arms and the number of entries into the open arms. In addition, both YTO and ZTO significantly decreased salivary corticosterone levels in mice (*p* ≤ 0.001). In summary, our results demonstrated that inhalation and transdermal administration of both YTO and ZTO showed anxiolytic effect on male ICR mice.

## 1. Introduction

Anxiety disorder is one of the most prevalent psychiatric disorders with typical symptoms including high blood pressure, chest pain, tachypnea, sweating, and irritability [1,2,3]. The most commonly used medications for the treatment of anxiety disorders are benzodiazepines (BZD) and selective serotonin reuptake inhibitors (SSRIs) [4]. However, long-term use of these substances may cause side effects such as amnesia, sleep disturbance, and drug dependence [4,5,6]. As an alternative therapy to traditional medicines, natural products derived from plants (e.g., essential oils) have better efficacy in the treatment of anxiety disorders and have less side effects [7,8,9].

Various essential oils have been found to have anxiolytic effects, such as lavender [10,11,12], rose [13,14], bergamot [15,16], and chamomile [17] oils. Especially, Silexan, a preparation extracted from *Lavandula angustifolia* L. flowers, has been confirmed to be effective in the treatment of anxiety disorders [18,19,20]. Essential oils of some indigenous plants used in folk medicine have been demonstrated to exhibit anxiolytic effect in animal experiments, such as the essential oils of *Coriandrum sativum* L. (coriander, a popular herb of the Apiaceae family), *Nectandra grandiflora* Ness (a native endemic tree from Southern Brazil), and *Cymbopogon citratus* (D.C.) Stapf (popularly known as lemongrass). Further studies have shown that the major components of essential oils also have anxiolytic effects, including alcohols, terpenes, aldehydes, and ketones [21].

Tobacco (*Nicotiana tabacum* Linn.), a species from the Solanaceae family native to South America, is widely cultivated in most parts of the world and is an essential non-food crop [22,23]. Tobacco leaf is a traditional herb used in folk medicine to treat mental health problems such as anxiety and depression [24]. As an important aromatic plant, tobacco contained richer compounds than other natural products [25]. Up to now, more than 3000 compounds have been identified in various tobacco species, including terpenoids, carotenoid degradation products, and various aromatics [26]. Moreover, some of these components are unique to tobacco [27].

Many studies have been devoted to assessing tobacco quality or analyzing tobacco aroma [22,26], while few studies have been conducted on the bioactivity of tobacco essential oils. It has been demonstrated through in vitro and animal experiments that tobacco essential oil and its major components have antibacterial [27,28,29,30,31], anti-inflammatory [32,33], antioxidant [33,34,35], antitumor [30], and neuroprotective effects [33]. Meanwhile, many essential oils or their components with the effects mentioned above have been found to have anxiolytic effects as well, such as oils of *Cananga odorata* (Lam.) Hook.f. & Thomson. (ylang-ylang) [36,37,38], coriander [3,39], *Rosmarinus officinalis* L. (rosemary) [40], and thymol [41]. However, relevant studies on the anxiolytic properties of tobacco essential oils have not been conducted so far.

Inhalation is the most frequently used route of essential oil administration in the animal behavior test. While in clinical and practical trials, essential oils are generally administered in a transdermal route. It has been demonstrated in several studies that transdermal administered essential oils are also able to produce anti-inflammatory effects on mice [42,43]. However, few studies evaluated the anxiolytic effects of essential oils by transdermal administration in animal studies. Hence, in the current study, the anxiolytic effect of essential oils on mice resulting from two administration routes, i.e., inhalation and transdermal administration, were differentiated.

It has been demonstrated that salivary corticosterone can be used as a biomarker to assess stress levels in rats [44,45,46]. Furthermore, salivary corticosterone sampling in mice was less invasive [47]. Therefore, in this study, salivary corticosterone levels were measured in mice to evaluate their level of anxiety.

The main objective of this study was to assess the anxiolytic effects of two tobacco essential oils administrated via inhalation and transdermal routes using two anxiety models in mice. In addition, the components of the oils were analyzed by GC/MS, and their acute oral and transdermal toxicity was evaluated. Salivary corticosterone levels were also measured in mice. To the best of our knowledge, this is the first report on the anxiolytic effect of tobacco essential oils.

## 2. Results

### 2.1. Chemical Composition of YTO and ZTO

**Figure 1:**
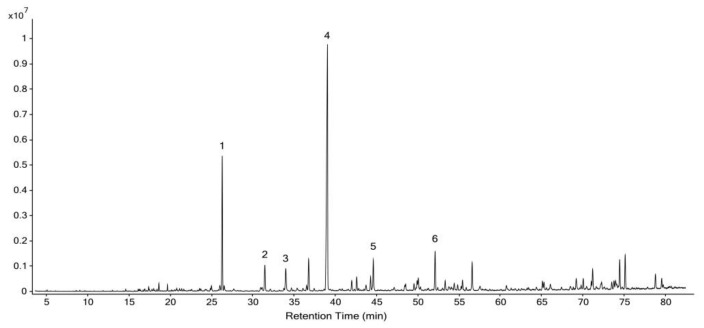
Total ion gas chromatogram of YTO. Significant peaks: 1 (±)-solanone; 2 trans-beta-damascenone; 3 nicotine; 4 neophytadiene; 5 3,4-dimethoxystyrene; 6 megastigmatrienone.

The composition of YTO and ZTO used in this study were analyzed by GC/MS. Table A1 lists the 22 compounds identified in YTO (Appendix A). As shown in Figure 1, six compounds with relative content (peak area %) higher than 2% were identified as neophytadiene (27.42% ± 0.46%), (±)-solanone (9.30% ± 0.16%), megastigmatrienone (3.12% ± 0.06%), 3,4-dimethoxystyrene (2.73% ± 0.04%), nicotine (2.35% ± 0.07%), and trans-beta-damascenone (2.32% ± 0.04%).

**Figure 2:**
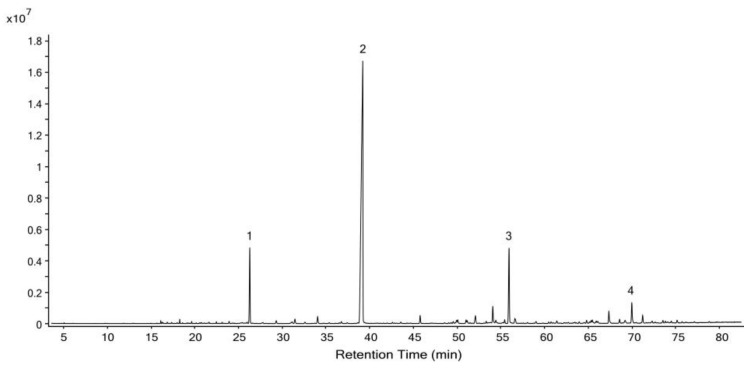
Total ion gas chromatogram of ZTO. Significant peaks: 1 (±)-solanone; 2 neophytadiene; 3 ethyl palmitate; 4 ethyl linolenate.

A total of 35 compounds were identified in ZTO and are listed in Table A2 (Appendix A). As shown in Figure 2, four compounds with relative content (peak area %) higher than 2% were identified as neophytadiene (59.82% ± 0.36%), ethyl palmitate (8.18% ± 0.07%), (±)-solanone (7.16% ± 0.05%), and ethyl linolenate (2.36% ± 0.00%).

Overall, the number of compounds was more abundant in ZTO than in YTO. A total of 13 components were identical in YTO and ZTO and are listed in Table 1. Among them, the compounds with obvious difference in peak area include neophytadiene (27.42% in YTO, 59.82% in ZTO), megastigmatrienone (3.12% in YTO, 1.05% in ZTO), trans-beta-damascenone (2.32% in YTO, 0.54% in ZTO), and ethyl palmitate (0.22% in YTO, 8.18% in ZTO). In addition, nicotine, the 5th richest compound in YTO, was not identified in ZTO.

### 2.2. Toxicity Evaluation of YTO and ZTO

#### 2.2.1. Acute Oral Toxicity Evaluation

The statistics of food consumption, weight gain, and weight gain ratio of each group in the acute oral toxicity experiment are shown in Table 2. One-way ANOVA analysis revealed a significant difference in food consumption between treatment groups (*F*(9,19) = 5.422, *p* < 0.001), while there was no significant difference in weight gain (*F*(9,19) = 0.584, *p* > 0.05) or weight gain ratio (*F*(9,19) = 0.945, *p* > 0.05). Food consumption was significantly increased in mice treated with 250 mg/kg ZTO (*p* < 0.05) compared with that in the saline group, and there were no statistically significant differences in food consumption among other groups.

On the first day of the oral toxicity test, mice dosed with 2000 mg/kg YTO showed abnormal conditions such as reduced activities and appetite, and one of them died on the second day. The other two mice recovered on the second day and showed no abnormal symptoms during the subsequent observation period. One of the mice treated with 250 mg/kg YTO was frightened during the gavage operation and then lost appetite and weight for the next two days, but it recovered from the third day and gained weight steadily during the observation period. No abnormalities were found in the other two mice in the same group. This resulted in a lower weight gain in the 250 mg/kg YTO group, but there was no significant difference compared to the saline group. Mice treated with 1000 mg/kg or less of YTO showed no abnormalities and increasing body weight within 14 days. The results indicated that oral administration of YTO under 1000 mg/kg is considered safe, while 2000 mg/kg of YTO has potential oral toxicity.

Mice given a single oral dose of 2000 mg/kg ZTO showed no mortality or other abnormal symptoms during the 14-day observation period, indicating that oral administration of ZTO up to 2000 mg/kg is safe. Mice treated with 250 mg/kg and 1000 mg/kg ZTO showed a significant increase in food consumption compared to the saline group. In addition, there was steady weight gain in all groups treated with ZTO. Taken together, these results suggest that ZTO has lower oral toxicity than YTO.

In summary, the results of the oral toxicity evaluation demonstrated that the concentrations of both YTO and ZTO used in the behavioral tests were considered safe.

#### 2.2.2. Acute Dermal Toxicity Evaluation

The statistics of the weight gain and weight gain ratio of female and male mice in the dermal toxicity experiments are shown in Table 3. One-way ANOVA analysis showed that the different treatments had no significant effect on weight gain (*F*(6,14) = 0.267, *p* > 0.05; *F*(6,14) = 1.301, *p* > 0.05) or weight gain ratio (*F*(6,14) = 0.311, *p* > 0.05; *F*(6,14) = 1.152, *p* > 0.05) in both female and male mice. The weight gain of female mice administered 2000 mg/kg YTO and ZTO was relatively lower compared to that of the control group. The weight gain of male mice administered 500 mg/kg and 1000 mg/kg ZTO was higher than that of the control group, while an opposite trend was observed for male mice dosed at 500 mg/kg of YTO and 2000 mg/kg of ZTO.

During the 14-day observation period, no pathological symptoms or mortality were observed in mice administered 2000 mg/kg of YTO and ZTO. We found that after the administration of 2000 mg/kg YTO, one female and one male showed temporary symptoms of discomfort, including piloerection, dullness, and apathy, but these symptoms disappeared the next day. Compared with the control group, the hair growth of the mice administered 2000 mg/kg YTO and ZTO was slower than other groups in the first four days, but the differences gradually diminished from the fifth day. There was no abnormal behavior observed in mice who received a dose of 1000 mg/kg and below.

According to the transdermal toxicity test results, 2000 mg/kg of YTO essential oil and ZTO essential oil are considered safe in terms of dermal toxicity. However, there was a possibility of affecting the rate of hair regrowth in mice. Higher concentrations of YTO essential oil may have potential dermal toxicity.

### 2.3. The Effects of Two Administration Routes on Control Groups

**Figure 3:**
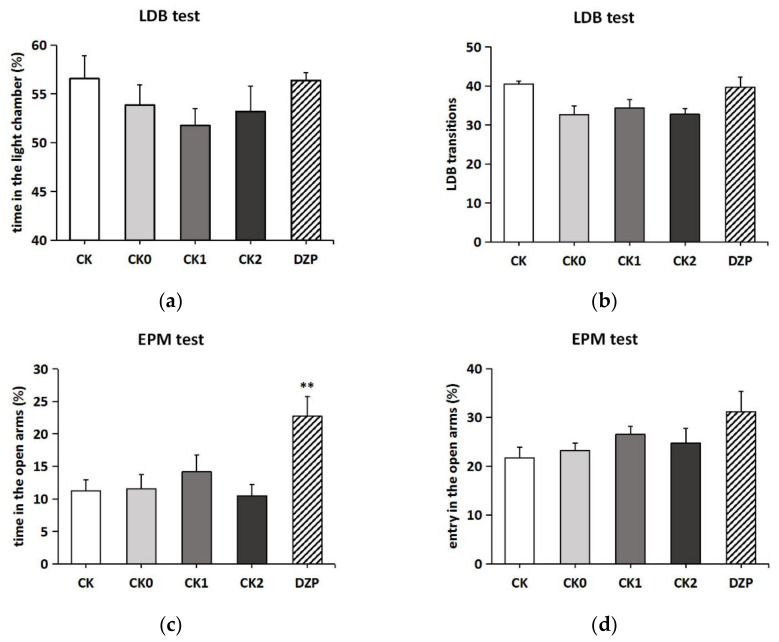
Anxiolytic effect of different administration methods on mice in the LDB and EPM test. (**a**) The time in the light chamber (%) in the LDB test. (**b**) The transitions in the LDB test. (**c**) The time in the open arms (%) in the EPM test. (**d**) The entry in the open arms (%) in the EPM test. (1) CK: blank control group; (2) CK0: inhalation apparatus control group; (3) CK1: olivia inhalation control group; (4) CK2: olivia transdermal control group; (5) DZP: diazepam (positive control drug) group. Values represent the mean ± SEM (*n* = 5~7). One-way ANOVA followed by a post hoc Tukey’s multiple comparison was used. ** *p* < 0.01, vs. CK group.

In the LDB test, one-way ANOVA showed that the different treatments had a significant effect on the parameters of transitions between different control groups (*F*(4,26) = 3.824, *p* < 0.05), while there was no significant effect on the percentage of time spent in the light chamber (LCT%, *F*(4,27) = 1.111, *p* > 0.05). As shown in Figure 3a,b, the manipulation of placing into the inhalation apparatus (CK0, CK1, CK2) decreased the LCT% and the number of transitions in the LDB compared to the blank control group (CK), while the LDB test parameters in the diazepam group (DZP) were comparable to that of the CK group. However, post hoc Turkey tests revealed no statistically significant differences in the LDB behavioral parameters between different groups. In the EPM test, different pretreatments also affected the percentage of time the mice spent in open arms (OT%, *F*(4,24) = 4.646, *p* < 0.01), but they had no effect on the percentage of the entry into open arms (OE%, *F*(4,26) = 2.037, *p* > 0.05). As shown in Figure 3c, the OT% of the DZP group was over 20%, which was significantly higher than that of the other control groups (<15%) (*p* < 0.01). Moreover, the DZP group also recorded the highest OE%, although there was no statistically significant difference (Figure 3d). In contrast to the LDB test results, the manipulation of placing in the inhalation apparatus did not affect the behavior of mice in the EPM test compared to the CK group. Inhalation administration (CK1) and transdermal administration (CK2) of olive oil brought no additional effects on the behavior of mice in the LDB and EPM test compared to the group that was simply placed in the inhalation apparatus for 30 min (CK0).

**Figure 4:**
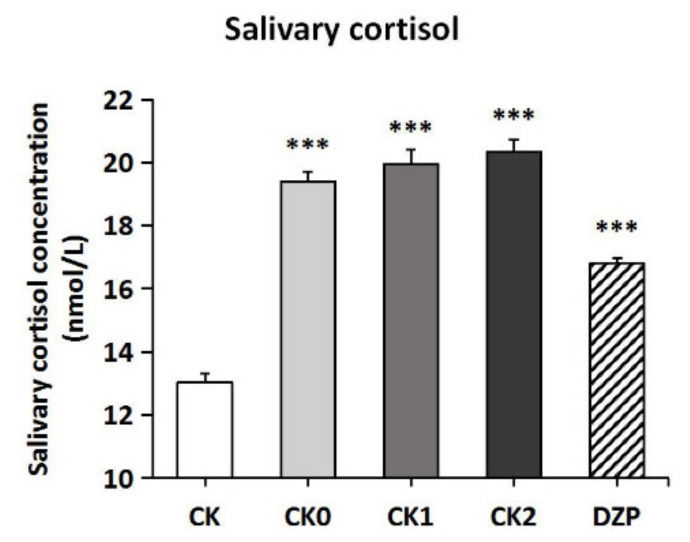
Changes of salivary cortisol content in mice under different administration methods. Values represent the mean ± SEM (*n* = 6~7). One-way ANOVA followed by a post hoc Tukey’s multiple comparison was used. *** *p* < 0.001, vs. CK group.

As shown in Figure 4, the different pretreatments significantly affected the salivary corticosterone content of mice (*F*(4,30) = 62.147, *p* < 0.001). Placing into the inhalation apparatus for 30 min significantly elevated salivary corticosterone levels in mice compared to the CK group (*p* < 0.001). On the other hand, DZP injection significantly reduced the anxiety level of mice compared to the CK0, CK1, and CK2 groups (*p* < 0.001). As for the results of the behavioral tests, inhalation or transdermal administration did not produce additional effects on salivary corticosterone levels in mice.

### 2.4. The Effect of YTO and ZTO on Mice in the LDB Test

**Figure 5:**
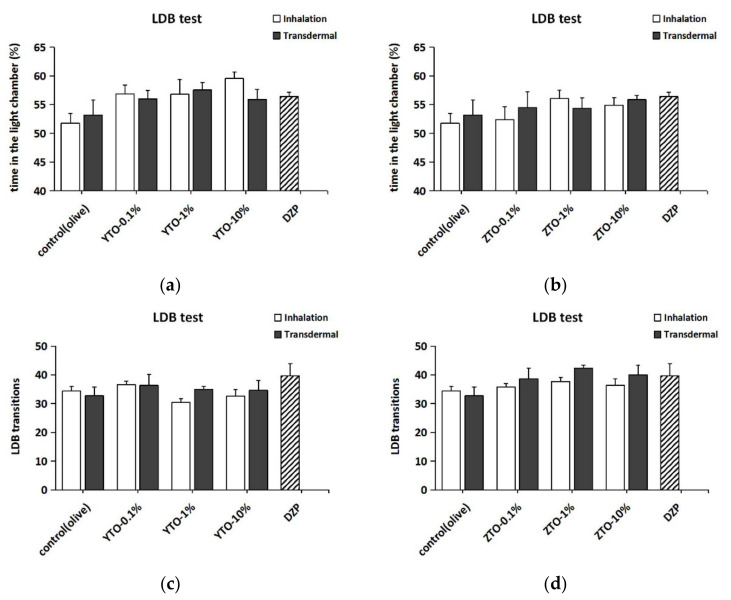
Anxiolytic effect of YTO and ZTO on mice in the LDB test. (**a**) The time in the light chamber (%) of mice treated with YTO. (**b**) The time in the light chamber (%) of mice treated with ZTO. (**c**) The transitions in the LDB test of mice treated with YTO. (**d**) The transitions in the LDB test of mice treated with ZTO. Values represent the mean ± SEM (*n* = 5~7). Two-way ANOVA followed by a post hoc Tukey’s multiple comparison was used.

In the LDB test, the administration of YTO and ZTO both reduced the level of anxiety in mice (Figure 5). As shown in Figure 5a,b, the administration of essential oils increased the LCT% of the mice. Nevertheless, the result of two-way ANOVA analysis showed that there was no significant difference in the behavior of mice with different treatments in LDB (LCT%: *F*(7,94) = 0.894, *p* > 0.05; transitions: *F*(7,86) = 1.964, *p* > 0.05). Furthermore, two-way ANOVA analysis revealed significant behavioral differences for the mice treated with different essential oil types in terms of both LCT% (*F*(1,94) = 5.712, *p* < 0.05) and transitions (*F*(1,86) = 11.462, *p* < 0.001). Comparing the anxiolytic effect of YTO and ZTO in the LDB test, we found that YTO enhanced LCT% more than ZTO (Figure 5a,b), while ZTO enhanced more transitions than YTO (Figure 5c,d). In addition, the two factors of treatment and type of essential oil played no interactive effect on LCT% (*F*(5,94) = 0.626, *p* > 0.05) and transitions (*F*(5,94) = 1.043, *p* > 0.05).

### 2.5. The Effect of YTO and ZTO on Mice in the EPM Test

**Figure 6:**
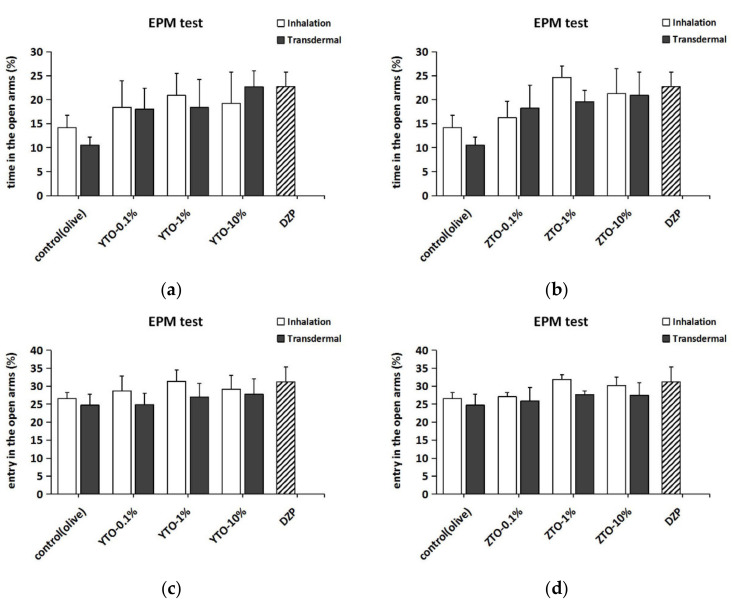
Anxiolytic effect of YTO and ZTO on mice in the EPM test. (**a**) The time in the open arms (%) of mice treated with YTO. (**b**) The time in the open arms (%) of mice treated with ZTO. (**c**) The entry in the open arms (%) of mice treated with YTO. (**d**) The entry in the open arms (%) of mice treated with ZTO. Values represent the mean ± SEM (*n* = 5~7). Two-way ANOVA followed by a post hoc Tukey’s multiple comparison was used.

In the EPM test, all concentrations of YTO and ZTO demonstrated anxiolytic effects (Figure 6). As shown in Figure 6a,b, the administration of YTO and ZTO all elevated the OT% of mice compared to that of the control group. Among them, the transdermal administration of 10% YTO and inhalation of 1% ZTO had the best effect on OT%, which was similar to the case with DZP. As shown in Figure 6c,d, the administration of both YTO and ZTO increased the OE%, with inhalation of 1% essential oil having the best effect. Nevertheless, the results of two-way ANOVA showed no significant differences in both OT% (*F*(7,88) = 0.750, *p* > 0.05) and OE% (*F*(7,86) = 0.963, *p* > 0.05) between the different treatment groups. Comparing the effects of the two administration methods on the behavior of mice in the EPM test, the post hoc Turkey test showed no significant difference between inhalation and transdermal administration. In addition, two-way ANOVA indicated that there were also no significant behavioral differences in EPM between mice treated with different essential oil types (OT%: *F*(1,88) = 0.047, *p* > 0.05; OE%: *F*(1,86) = 0.015, *p* > 0.05). As with the LDB test, treatment and essential oil type factors also had no interactive effect on OT% (*F*(5,88) = 0.120, *p* > 0.05) and OE% (*F*(5,86) = 0.047, *p* > 0.05).

### 2.6. Changes of Salivary Corticosterone in Mice after YTO and ZTO Administration

**Figure 7:**
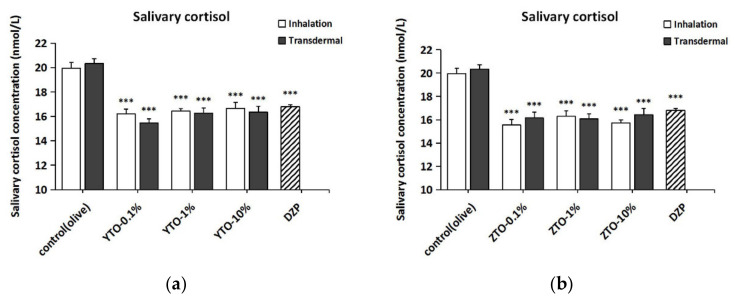
Changes in the content of salivary corticosterone in mice after YTO and ZTO administration. (**a**) YTO groups. (**b**) ZTO groups. Values represent the mean ± SEM (*n* = 6~7). Two-way ANOVA followed by a post hoc Tukey’s multiple comparison was used. *** *p* < 0.001, vs. CK group.

The results of two-way ANOVA indicated that the different treatments significantly affected salivary corticosterone levels in mice (*F*(7,103) = 4.880, *p* < 0.001). As shown in Figure 7a,b, the administration of all concentrations of YTO and ZTO significantly reduced salivary corticosterone levels in mice compared to the control group (*p* < 0.001). The salivary corticosterone levels were generally lower in all essential oil administered groups than in the DZP group, although the difference was not significant. Moreover, according to the two-way ANOVA, there was no significant difference in the salivary cortisol content of mice between the different essential oil types (*F*(1,103) = 1.636, *p* > 0.05), and no interaction effect was exerted by the treatment and essential oil type factors (*F*(5,103) = 0.508, *p* > 0.05).

## 3. Discussion

Despite being an ancient herb, tobacco has been controversial in recent years due to its proven health hazards [24,48]. It is known that cigarette smoke contains harmful substances (such as nicotine) and can cause several smoking-related diseases [49,50]. Nevertheless, it cannot be ignored that tobacco is abundant in bioactive substances and is a natural material of great research value [26,27]. Therefore, it is necessary to develop more beneficial uses of tobacco. In previous studies, tobacco essential oil was found to have antibacterial [27,28,29,30,31], anti-inflammatory [32,33], antioxidant [33,34,35], and antitumor [30] effects as well as neuroprotective potential [33]. However, no studies have reported the anxiolytic effect of tobacco essential oil. In the current study, we evaluated the anxiolytic effects of Yunnan and Zimbabwe tobacco essential oils on ICR mice using LDB and EPM paradigms and by referring to salivary corticosterone levels. This work provides evidence that both inhalation and transdermal administration of two tobacco oils induce anxiolytic-like effects, which complements the potential beneficial uses of tobacco oils and expands their possible applications.

In this study, we identified the components of YTO and ZTO by GC/MS and compared them. Among the identical constituents of both essential oils, the highest relative contents were neophytadiene and solanone, which were the primary sources of the unique aroma of tobacco [29]. In addition, megastigmatrienone and trans-beta-damascenone also play an essential role in the aroma and flavor of tobacco [29]. Notably, no nicotine was identified in the ZTO, which is inconsistent with previous studies [51,52]. This compositional difference can be explained by differences in the origin of the plant material and the processing conditions of the essential oils [26]. Previous studies have indicated that the anxiolytic effects of essential oils could be related to their main chemical composition [21,38,53,54]. Accordingly, we speculate that the anxiolytic effects of YTO and ZTO could be attributed to the several major aroma components mentioned above. Neophytadiene has been proven to have antioxidant and anti-inflammatory effects [32], but its anxiolytic effects have not been reported in the literature. In future work, we could conduct further studies on the anxiolytic efficacy of the major components of tobacco essential oil.

The results of the toxicity evaluation showed that YTO had significantly higher oral toxicity than ZTO. Moreover, YTO could have potential transdermal toxicity, while ZTO did not. Among the shared components, the relative content of megastigmatrienone (irritate, harmful if swallowed) [55] and trans-beta-damascenone (causes skin irritation) [56] was higher in YTO than in ZTO. Furthermore, nicotine with a relative content of 2.13% in YTO was not detected in ZTO, while ethyl palmitate (no toxicity was reported) [57], the second-highest relative content in ZTO, was not identified in YTO. In addition, given that ZTO showed low toxicity, we believe that the use of ZTO instead of cigarettes could reduce exposure to some tobacco toxicants. In this study, the toxicity of YTO and ZTO was evaluated to ensure that the concentrations of essential oils used in the anxiolytic test were safe. Therefore, a comprehensive toxicity evaluation of YTO and ZTO was not conducted. In future studies, it is necessary to conduct a more systematic evaluation of the toxicity of tobacco essential oils, so as to provide a detailed reference for the safe application of tobacco essential oil.

The results of the anxiety level test in the control group showed that the manipulation of transdermal and inhalation administration itself did not exert additional effects on the mice. However, we found that the manipulation of placing into the inhalation apparatus induced anxiety in mice, and DZP could mitigate this effect. This is strongly supported by the results of salivary corticosterone analysis. The aversion of rodents to unfamiliar environments [58] could explain the effects induced by the inhalation apparatus. It is suggested that the effects of the inhalation device on the animals need to be considered when assessing the anxiolytic efficacy of inhaled essential oils. On the other hand, placing into the inhalation device could also be seen as providing an initial model of the anxiety state. However, this opinion requires specialized experiments to verify, as we could not determine whether this anxiety effect is related to the residence time in the device.

Furthermore, the parameters of the blank control group were similar to that of the DZP group in the LDB test, but the results in the EPM test were significantly lower than those of the DZP group. This result results from the mechanistic differences between the two behavioral models [3,59]. The LDB test is based on the natural aversion of rodents to bright places [60]. In contrast, the EPM test is based on the aversion to open space, which creates a conflict between exploration and aversion to high altitude places [58]. Therefore, we should consider this difference when analyzing the results of behavioral tests on tobacco essential oils.

In contrast to clinical studies, transdermal administration has rarely been used in animal experiments to assess the anxiolytic efficacy of essential oils [21]. This could be attributed to the ability of essential oils to act in a fast and significant way through the olfactory pathway [10]. However, considering that skin application is also a wildly used administration route of essential oils in practical applications, it is necessary to evaluate whether essential oils can exert anxiolytic effects on mice through transdermal administration. In the current study, we demonstrated that two tobacco essential oils also produced anxiolytic effects when administered through the skin, and the effects were not significantly different from those of inhalation administration. Our results provide a reference for the development of new application routes of essential oil in animal models of anxiety. It should be noted that the transdermal administration method we used in this study did not disrupt the olfactory sensation of the mice. Further experiments are required to determine the anxiolytic effect of tobacco essential oil through transdermal absorption. For instance, the deprivation of olfaction does not affect the anxiolytic efficacy of lavender essential oil [61], but its main component, linalool, does not exert anxiolytic effects in the absence of olfaction [62].

However, one drawback of the transdermal administration method is that mice in the transdermal administration group were observed to have significantly more grooming behavior in behavioral tests. In the LDB test, the mice in the transdermal administration group spent more time grooming than that in the inhalation group. Furthermore, we also observed in the EPM test that the mice treated with transdermal administration spend more time grooming, which is often at the innermost end of the closed arm. It is possible that this behavior influenced the LCT% and OT% of mice in the transdermal administration group. In low-stress states, grooming is a routine in mice, whereas in stressful situations, grooming behavior becomes frequent and rapid [63]. Previous studies have shown that essential oils and anxiolytics are usually able to decrease the grooming behavior of animals in open field tests [16,64]. Therefore, we were unable to determine how the increased grooming behavior associated with transdermal administration would affect the results of behavioral tests. However, transdermal administration showed no significant differences in the testing of salivary corticosterone.

The results of behavioral tests in mice showed that YTO and ZTO at 0.1%, 1%, and 10% concentrations exhibited anxiolytic-like effects in both the LDB test and the EPM test. Overall, YTO and ZTO showed more significant anxiolytic effects in the EPM test than in the LDB test, which can be explained by the differences between the models mentioned previously. Furthermore, given that rodents are exquisitely sensitive to the luminance of the environment [65], and the LDB model has a light source different from the EPM model, the experimental environment may also affect the behavior of mice in both models. In addition, salivary corticosterone levels were significantly reduced in mice treated with essential oils compared to the control group, which powerfully demonstrates the anxiolytic effect of YTO and ZTO. Our study also confirmed that salivary corticosterone is a valid biomarker for assessing anxiety levels in mice [47]. Previous studies have shown individual differences in behavioral performance in mice [66]; thus, we suggest that salivary corticosterone could be used as a complementary physiological indicator to assess the anxiolytic efficacy of essential oils.

Comparing the anxiolytic effects of YTO and ZTO, we found significant differences between the different essential oil varieties in the LDB test, while there were no significant differences in the EPM test and salivary corticosterone levels. In the LDB test, YTO showed a higher elevation in LCT% than ZTO but a lower number of transitions than ZTO. It could be related to the composition of the two essential oils. The anxiolytic effect of the two essential oils was not significantly different, suggesting that the shared components (such as neophytadiene and solanone, or other aroma substances) play a role in anxiolytic efficacy. However, the toxic substances in YTO may have reduced the mice’s motility, thus decreasing the parameters of transitions in the LDB test. However, this could not explain the LCT% improvement. Hence, mechanisms of the anxiolytic effects of YTO and ZTO need to be further explored in the future for a better understanding of the reasons underlying these differences.

## 4. Materials and Methods

### 4.1. Plant Material and Essential Oil Extraction

Two kinds of tobacco (*Nicotiana tabacum* Linn.) essential oils were obtained from the leaves of K326 tobacco produced in Yunnan (YTO) and “Jinyin No.1” tobacco produced in Zimbabwe (ZTO), respectively. Normal-pressure steam distillation is used for extraction. First, the dried tobacco leaves were pulverized through a 40-mesh sieve to obtain the tobacco powder. Next, one Kg tobacco powder and 10 kg water were added to a 20 L steam distillation device and distilled in water for 6 h. Finally, the essential oil fraction was collected, dried over anhydrous sodium sulfate, and filtered to obtain brown tobacco leaf essential oil. On average, 0.2 g YTO or 0.3 g ZTO could be extracted from 1 kg of dry tobacco leaves.

### 4.2. Animals

ICR mice (6–8 weeks) were purchased from the Zhejiang Vital River Laboratory Animal Technology Co., LTD., China. The animals were housed in polypropylene cages (3 animals/cage or 6 animals/cage) and maintained in a controlled environment (25 ± 1 °C temperature, 50 ± 5% relative humidity, and under a 12 h/12 h light/dark cycle) with free food and water. At the beginning of experiment, the weight of the male mice was 22–30 g and the female mice was 20–25 g. All animals were acclimatized for one week before the experiment.

### 4.3. Chemicals and Treatments

The YTO and ZTO (provided by Shanghai Huabao Food Flavours & Fragrances Co., Ltd., Shanghai, China) were diluted with olive oil to produce different concentrations (0.1%, 1%, and 10%, *v*/*v*) before experiments. Diazepam (DZP) (Shanghai Xudong Haipu Pharmaceutical Co., LTD., Shanghai, China), diluted with saline, was used as a positive control drug (1 mg/kg). Mice were given an intraperitoneal dose of 0.1 mL/10 g body weight of the diluted DZP 30 min before behavioral tests. Every effort was made to minimize the number of animals and to reduce their suffering.

### 4.4. Gas Chromatography/Mass Spectrometry (GC/MS) Analysis of the Tobacco Essential Oil

The composition of the YTO and ZTO was determined by GC/MS (Agilent 7890B-5977A GC-MS, USA). The chromatographic separation was conducted on a capillary column (DB-WAX, 30 m × 250 μm × 0.25 μm). The GC conditions: carrier gas, helium (99.999%, 1 mL/min); split ratio, 30:1; column temperature, 50 °C for 3 min, then increased to 120 °C at 4 °C/min, maintained for 10 min, and increased to 220 °C at 2 °C/min, then kept constant at 220 °C for 2 min. The MS conditions: inlet line temperature, 260 °C; source temperature, 230 °C; mass spectra, electron impact (EI), 70 eV.

The individual component was identified by referring to the NIST14 mass spectral library, NIST Chemistry WebBook SRD 69 [67] and the FFNSC (Flavors and Fragrances of Natural and Synthetic Compounds) Library. The identification results were verified comparing the retention (Kovat’s) indices and mass spectra of the identified components with the corresponding compounds in the reference database.

### 4.5. Inhalation Apparatus

Odor exposure was performed using an olfactory inhalation apparatus as described by Nan Zhang et al. [38]. The inhalation apparatus (65 × 65 × 45 cm^3^) was made of stainless steel with a clear lid. Eight perforated walls separated the apparatus into four small chambers (25 × 25 × 30 cm^3^) and formed a cross-shaped area in the center where an anhydrous aromatherapy apparatus (MEETA-NC11, Fuzhou, China) was placed. Mice were individually placed in the chamber for 30 min of odor exposure before behavioral tests.

### 4.6. Acute Toxicity of Essential Oil

#### 4.6.1. Acute Oral Toxicity: (OECD Guidelines-425, 2008)

The oral toxicity of two types of tobacco essential oils was assessed according to the OECD guidelines 425 and the Chinese Toxicological test methods for pesticide registration. Female ICR mice were divided into ten groups (*n* = 3) and fasted and water-deprived the night before the experiment. Eight groups of mice (4 groups for each essential oil) were pretreated intragastrically with 100 uL of essential oil diluted in olive oil (*w*/*v*) at the following doses: 250, 500, 1000, and 2000 mg/kg body weight. Mice in the saline group were intragastrically administered an equal volume of 0.9% saline, and the Control group received olive oil. After oral treatment, the animals were observed continuously at 1 h, 3 h, and 24 h daily for 14 days. The body weight and food intake of the animals, as well as abnormal behaviors (e.g., tremor, convulsions, salivation, diarrhea, lethargy, coma, bleeding, and other symptoms) were recorded daily.

#### 4.6.2. Acute Dermal Toxicity: (OECD Guidelines-402, 2017)

The dermal toxicity of two types of tobacco essential oils was assessed according to the OECD guidelines 402 and the Chinese Toxicological test methods for pesticide registration. First, ICR mice were divided into seven groups (*n* = 10), half male and half female. A 4 cm^2^ area of hair on the back of the mice was shaved 24 h before the start of the experiment. Then, 100 uL of essential oil diluted in olive oil (*w*/*v*) (500, 1000, and 2000 mg/kg body weight) was evenly smeared on the back of mice. Then, the mice were fixed with non-irritating gauze and tape, preventing animals from licking. After 24 h of application, the drugs were washed out using distilled water, and the status of the animals was observed at 30 min, 4 h, and 24 h after drug administration. The animal’s performance and condition were recorded individually and comprehensively daily for 14 consecutive days. The bodyweight of animals was recorded pre-dose, post-dose, and on day 7 and day 14 of observation.

### 4.7. Behavioral Tests

#### 4.7.1. Light–Dark Box Test (LDB)

The light–dark box (Shanghai XinTuan Information Technology Co., Ltd., Shanghai, China) consisted of two compartments of different sizes. The light box was a larger chamber with a white lid (27 × 18 × 18 cm^3^) covered by white impermeable glue, whereas the dark box was a chamber with a black lid (18 × 18 × 18 cm^3^) covered by black impermeable glue. The light chamber was equipped with a 400 lux illuminating light at the bottom. The two rooms were separated by a wall with an open door (5 × 5 cm^2^). The top part of the box was fitted with a micro-camera used to detect the animal’s position and movement.

The LDB model is established based on the animal’s paradoxical psyche of having a natural aversion to bright places and a curious, exploratory disposition to the environment [58]. At the beginning of the experiment, the mice were placed in the light chamber and allowed to explore the entire device freely. The behavioral parameters of the mice were recorded by camera for 5 min. Two anxiety parameters were recorded in the LDB test: the percentage of time spent in the light chamber (LCT% = time in the light chamber/total time × 100%) and the number of transitions between two chambers [54].

#### 4.7.2. Elevated Plus Maze Test (EPM)

The elevated plus maze ((Shanghai XinTuan Information Technology Co., Ltd., Shanghai, China) consisted of four arms (open arms: 30 × 6 cm^2^; closed arms: 35 × 6 × 15 cm^3^) placed in a cross shape, with a square platform (6 × 6 cm^2^) formed in the center. The whole maze was raised to a height of 55 cm. A USB camera (Aoni C11, Guangzhou, China) was installed above the maze to record the position and movement of the mice. At the beginning of the test, mice were placed on the central platform facing one of the open arm and were allowed to explore the entire maze freely. The behavioral parameters of the mice were recorded for 5 min, including: (1) the time spent in open and closed arms (OT and CT); (2) the number of entries into open and closed arms (OE and CE). The percentage of the entry into open arms (OE% = OE/(OE + CE) × 100%) and time spent in open arms (OT% = OT/(OT + CT) × 100%) were calculated and considered as anxiety parameters [54]. A single entry was considered when the mice had all paws placed into one arm.

### 4.8. Collection and Examination of Salivary Corticosterone

Saliva samples were obtained by placing a sterilized cotton swab in the mouth of the mice for more than 1 min immediately after the behavioral tests and were sealed in a centrifuge tube and stored at −20 °C. The level of salivary corticosterone was determined using an enzyme-linked immunosorbent assay (ELISA) kit (Shanghai Hengyuan Biotechnology Co., Ltd., Shanghai, China).

### 4.9. Experimental Procedures of Anxiolytic Effect

Male ICR mice were divided into 17 groups (*n* = 7). The controls were pretreated as follows before the behavioral tests: (1) Blank control group (CK): without any treatment; (2) Inhalation apparatus control group (CK0): without any reagent treatment, placed into the olfactory box for 30 min before behavioral tests; (3) Inhalation control (CK1) and transdermal control group (CK2): inhalation of olive oil, or applied 100 uL of olive oil transdermally on the back and placed in the inhalation apparatus for 30 min; (4) Positive control group (diazepam group, DZP): i.p. application of 1.0 mg/kg of diazepam solution before behavioral tests.

Essential oil administration groups were treated as follows. The inhalation administration group inhaled 0.1%, 1%, and 10% concentrations of essential oils for 30 min in an inhalation apparatus. For the transdermal administration group, 100 uL of essential oils at different concentrations (0.1%, 1%, and 10%) were applied on the back and put into the inhalation apparatus for 30 min.

On the day of the behavioral tests, the mice were habituated in the experimental environment in advance for 2 h. Except for the CK group, the mice in other groups were put into the inhalation apparatus for 30 min after reagent treatment, followed by 5 min of LDB test and 5 min of EPM test, and the saliva was collected immediately after the behavioral tests. Considering the circadian rhythm of the mice, all behavioral experiments were performed between 1:00 PM and 6:00 PM.

### 4.10. Statistical Analyses

The behavior of the animals in the EPM and LDB was analyzed with the Super Maze video tracking system (Shanghai XinRuan Information Technology Co., Ltd., Shanghai, China). One-way ANOVA followed by a post hoc Tuckey test was used to analyze the toxicity and anxiolytic effects of YTO and ZTO. Two-way ANOVA followed by a post hoc Tukey’s multiple comparisons test was used to determine the effect of essential oil types and the experimental treatments on behavior and salivary cortisol level of mice. All statistical analyses were conducted using the IBM SPSS Statistics 23 software.

## 5. Conclusions

In conclusion, the results of our study demonstrated that YTO and ZTO possessed anxiolytic effects in the LDB and EPM tests. Both inhalation and transdermal administration of the tobacco essential oils could exert anxiolytic effects. To the best of our knowledge, there are no reports on the anxiolytic effects of tobacco essential oil. Therefore, we moved a step forward in the enlightenment of applications of tobacco essential oils. In addition, the anxiolytic efficacy of the main compounds of the two tobacco essential oils still needs to be evaluated in future studies to understand the anxiolytic mechanism of tobacco essential oils in depth.

## Figures and Tables

**Table 1:** Comparison of chemical composition of YTO and ZTO.

No.	Compound	Peak Area in YTO (%)	Peak Area in ZTO (%)
1	Neophytadiene	27.42 ± 0.46	59.82 ± 0.36
2	(±)-Solanone	9.30 ± 0.16	7.16 ± 0.05
3	Megastigmatrienone	3.12 ± 0.06	1.05 ± 0.00
4	trans-beta-Damascenone	2.32 ± 0.04	0.54 ± 0.00
5	Phytone	0.53 ± 0.01	0.23 ± 0.01
6	Benzaldehyde	0.42 ± 0.01	0.05 ± 0.00
7	Benzyl alcohol	0.38 ± 0.01	0.10 ± 0.00
8	Methyl palmitate	0.29 ± 0.01	1.88 ± 0.05
9	Linalool	0.27 ± 0.01	0.13 ± 0.01
10	Ethyl palmitate	0.22 ± 0.00	8.18 ± 0.07
11	Phenylethyl alcohol	0.21 ± 0.00	0.10 ± 0.00
12	trans-beta-Ionone	0.18 ± 0.00	0.11 ± 0.01
13	5-Methyl furfural	0.07 ± 0.01	0.05 ± 0.00

**Table 2:** Evaluation of food consumption and weight gain of animals from control and YTO and ZTO treated groups (oral).

Group		Food Consumed (g)	Weight Gain (g)	Weight Gain Ratio (%)
Saline		52.60 ± 1.31	4.13 ± 0.32	21.22 ± 1.52
Control (olive oil)		51.03 ± 0.60	3.63 ± 0.69	17.98 ± 3.95
YTO	250 mg/kg	49.80 ± 0.56	2.93 ± 0.43	12.16 ± 0.15
	500 mg/kg	55.50 ± 1.53	3.93 ± 1.02	18.49 ± 5.11
	1000 mg/kg	57.73 ± 1.84	4.90 ± 0.78	24.29 ± 4.03
	2000 mg/kg	52.47 ± 2.78	3.90 ± 0.00	19.93 ± 1.27
ZTO	250 mg/kg	61.57 ± 2.64 *	4.00 ± 0.93	19.09 ± 4.35
	500 mg/kg	51.97 ± 1.94	3.80 ± 0.61	18.73 ± 2.74
	1000 mg/kg	58.63 ± 0.83	3.73 ± 0.33	18.42 ± 1.73
	2000 mg/kg	52.63 ± 1.72	4.10 ± 0.36	19.66 ± 1.34

Values represent the mean ± SEM (*n* = 2~3). One-way ANOVA followed by a post hoc Tukey’s multiple comparison was used. * *p* < 0.05, vs. saline group.

**Table 3:** Parameters of weight gain of animals from control and YTO and ZTO treated groups (dermal).

Gender	Group		Weight Gain (g)	Weight Gain Ratio (%)
Female	Control (olive oil)		3.17 ± 1.20	15.57 ± 5.78
	YTO	500 mg/kg	2.33 ± 0.17	11.07 ± 1.02
		1000 mg/kg	2.67 ± 1.88	13.06 ± 9.33
		2000 mg/kg	1.83 ± 1.48	8.57 ± 6.75
	ZTO	500 mg/kg	2.50 ± 0.29	11.42 ± 1.53
		1000 mg/kg	2.33 ± 1.20	10.82 ± 5.53
		2000 mg/kg	1.33 ± 0.60	6.12 ± 2.69
Male	Control (olive oil)		7.17 ± 0.88	32.60 ± 4.79
	YTO	500 mg/kg	5.83 ± 0.44	25.76 ± 2.03
		1000 mg/kg	6.83 ± 0.17	30.63 ± 1.19
		2000 mg/kg	7.00 ± 1.26	31.82 ± 6.19
	ZTO	500 mg/kg	9.17 ± 1.30	40.54 ± 4.52
		1000 mg/kg	8.67 ± 1.48	38.21 ± 6.34
		2000 mg/kg	5.33 ± 1.97	24.16 ± 9.46

Values represent the mean ± SEM (*n* = 3). One-way ANOVA followed by a post hoc Tukey’s multiple comparison was used.

## Data Availability

The data of essential oil components presented in this study are available in the Appendix A.

## Appendix A

**Table A1:** Compounds in YTO identified by GC/MS.

No.	Compound	CAS	Retention (Kovat’s) Indices		Peak Area (%) *
			RI_exp_	RI_ref_	
1	1-Butanol, 3-methyl-	000123-51-3	1209	1209	0.05 ± 0.00
2	5-Hepten-2-one, 6-methyl-	000110-93-0	1334	1338	0.04 ± 0.00
3	3-Hexen-1-ol, (Z)-	000928-96-1	1385	1386	0.07 ± 0.00
4	Benzaldehyde	000100-52-7	1514	1520	0.42 ± 0.01
5	Linalool	000078-70-6	1548	1547	0.27 ± 0.01
6	5-Methyl furfural	000620-02-0	1565	1570	0.07 ± 0.01
7	4-Methoxystyrene	000637-69-4	1664	1684	0.16 ± 0.01
8	(±)-Solanone	054868-48-3	1720	1738	9.30 ± 0.16
9	Benzene, 1,4-dimethoxy-	000150-78-7	1724	1727	0.51 ± 0.01
10	trans-beta-Damascenone	023726-93-4	1807	1810	2.32 ± 0.04
11	Dihydro-beta-ionone	017283-81-7	1817	1842	0.21 ± 0.01
12	Nicotine	000054-11-5	1845	1863	2.35 ± 0.07
13	Benzyl alcohol	000100-51-6	1866	1870	0.38 ± 0.01
14	Phenylethyl alcohol	000060-12-8	1898	1906	0.21 ± 0.00
15	trans-beta-Ionone	000079-77-6	1918	1917	0.18 ± 0.00
16	Neophytadiene	000504-96-1	1925	1922	27.42 ± 0.46
17	3,4-Dimethoxystyrene	006380-23-0	2022	2027	2.73 ± 0.04
18	Benzofuran, 5-methoxy-6,7-dimethyl-	035355-35-2	2094	NA	0.38 ± 0.01
19	Phytone	000502-69-2	2116	2110	0.53 ± 0.01
20	Megastigmatrienone	038818-55-2	2169	NA	3.12 ± 0.06
21	Methyl palmitate	000112-39-0	2209	2208	0.29 ± 0.01
22	Ethyl palmitate	000628-97-7	2249	2251	0.22 ± 0.00

* Values represent the mean ± SEM (*n* = 3); RI_exp_: experimental value; RI_ref_: reference values.

**Table A2:** Compounds in ZTO identified by GC/MS.

No.	Compound	CAS	Retention (Kovat’s) Indices		Peak Area (%) *
			RI_exp_	RI_ref_	
1	Ethyl octanoate	000106-32-1	1433	1435	0.15 ± 0.00
2	Ethyl sorbate	002396-84-1	1503	1501	0.23 ± 0.00
3	Benzaldehyde	000100-52-7	1513	1520	0.05 ± 0.00
4	2-Nonenal, (E)-	018829-56-6	1529	1534	0.04 ± 0.00
5	Ethyl nonanoate	000123-29-5	1534	1531	0.06 ± 0.00
6	Linalool	000078-70-6	1548	1547	0.13 ± 0.01
7	5-Methyl furfural	000620-02-0	1565	1570	0.05 ± 0.00
8	2,6-Nonadienal, (E,Z)-	000557-48-2	1579	1584	0.05 ± 0.00
9	Ethyl decanoate	000110-38-3	1632	1638	0.12 ± 0.00
10	Diethyl succinate	000123-25-1	1669	1675	0.19 ± 0.00
11	(±)-Solanone	054868-48-3	1720	1738	7.16 ± 0.05
12	Ethyl phenylacetate	000101-97-3	1771	1783	0.34 ± 0.01
13	beta-Damascone	035044-68-9	1802	1824	0.31 ± 0.04
14	trans-beta-Damascenone	023726-93-4	1807	1810	0.54 ± 0.00
15	Naphthalene, 2-methyl-	000091-57-6	1824	1852	0.18 ± 0.00
16	Geranylacetone	003796-70-1	1846	1840	0.82 ± 0.00
17	Benzyl alcohol	000100-51-6	1866	1870	0.10 ± 0.00
18	Phenylethyl alcohol	000060-12-8	1898	1906	0.10 ± 0.00
19	trans-beta-Ionone	000079-77-6	1918	1917	0.11 ± 0.01
20	Neophytadiene	000504-96-1	1928	1922	59.82 ± 0.36
21	Methyl myristate	000124-10-7	2002	2005	0.15 ± 0.00
22	Ethyl myristate	000124-06-1	2044	2049	0.82 ± 0.00
23	Methyl pentadecanoate	007132-64-1	2106	2108	0.08 ± 0.01
24	Phytone	000502-69-2	2117	2110	0.23 ± 0.01
25	Ethyl pentadecanoate	041114-00-5	2146	2148	0.31 ± 0.00
26	Cembrene	001898-13-1	2149	2181	0.26 ± 0.01
27	Megastigmatrienone	038818-55-2	2168	NA	1.05 ± 0.00
28	Methyl palmitate	000112-39-0	2210	2208	1.88 ± 0.05
29	Ethyl palmitate	000628-97-7	2250	2251	8.18 ± 0.07
30	Ethyl heptadecanoate	014010-23-2	2352	2349	0.17 ± 0.01
31	Ethyl stearate	000111-61-5	2455	2451	0.33 ± 0.02
32	Ethyl oleate	000111-62-6	2471	2471	0.45 ± 0.05
33	Ethyl linoleate	000544-35-4	2518	2521	1.44 ± 0.02
34	Methyl linolenate	000301-00-8	2549	2571	0.44 ± 0.00
35	Ethyl linolenate	001191-41-9	2584	2591	2.36 ± 0.00

* Values represent the mean ± SEM (*n* = 3); RI_exp_: experimental value; RI_ref_: reference values.
